# Application of an Interpretable Machine Learning Model to Predict Lymph Node Metastasis in Patients with Laryngeal Carcinoma

**DOI:** 10.1155/2022/6356399

**Published:** 2022-11-12

**Authors:** Menglong Feng, Juhong Zhang, Xiaoqing Zhou, Hailan Mo, Lifeng Jia, Chanyuan Zhang, Yaqin Hu, Wei Yuan

**Affiliations:** ^1^Chongqing Medical University, Chongqing 400016, China; ^2^Chongqing Institute of Green and Intelligent Technology, Chinese Academy of Sciences, Chongqing 400714, China; ^3^Chongqing School, University of Chinese Academy of Sciences, Chongqing 400714, China; ^4^Department of Otolaryngology & Head and Neck, Chongqing General Hospital, Chongqing 401147, China

## Abstract

**Objectives:**

A more accurate preoperative prediction of lymph node metastasis (LNM) plays a decisive role in the selection of treatment in patients with laryngeal carcinoma (LC). This study aimed to develop a machine learning (ML) prediction model for predicting LNM in patients with LC.

**Methods:**

We collected and retrospectively analysed 4887 LC patients with detailed demographical characteristics including age at diagnosis, race, sex, primary site, histology, number of tumours, T-stage, grade, and tumour size in the National Institutes of Health (NIH) Surveillance, Epidemiology, and End Results (SEER) database from 2005 to 2015. A correlation analysis of all variables was evaluated by the Pearson correlation. Independent risk factors for LC patients with LNM were identified by univariate and multivariate logistic regression analyses. Afterward, patients were randomly divided into training and test sets in a ratio of 8 to 2. On this basis, we established logistic regression (LR), k-nearest neighbor (KNN), support vector machine (SVM), extreme gradient boosting (XGBoost), random forest (RF), and light gradient boosting machine (LightGBM) algorithm models based on ML. The area under the receiver operating characteristic curve (AUC) value, accuracy, precision, recall rate, F1-score, specificity, and Brier score was adopted to evaluate and compare the prediction performance of the models. Finally, the Shapley additive explanation (SHAP) method was used to interpret the association between each feature variable and target variables based on the best model.

**Results:**

Of the 4887 total LC patients, 3409 were without LNM (69.76%), and 1478 had LNM (30.24%). The result of the Pearson correlation showed that variables were weakly correlated with each other. The independent risk factors for LC patients with LNM were age at diagnosis, race, primary site, number of tumours, tumour size, grade, and T-stage. Among six models, XGBoost displayed a better performance for predicting LNM, with five performance metrics outperforming other models in the training set (AUC: 0.791 (95% CI: 0.776–0.806), accuracy: 0.739, recall rate: 0.638, F1-score: 0.663, and Brier score: 0.165), and similar results were observed in the test set. Moreover, the SHAP value of XGBoost was calculated, and the result showed that the three features, T-stage, primary site, and grade, had the greatest impact on predicting the outcomes.

**Conclusions:**

The XGBoost model performed better and can be applied to forecast the LNM of LC, offering a valuable and significant reference for clinicians in advanced decision-making.

## 1. Introduction

Laryngeal carcinoma (LC) is one of the most common primary malignancies in the head and neck region, with an increasing incidence annually [1]. The incidence and mortality of LC have reached 2.76/10^5^ and 1.66/10^5^ in the world, which pose a serious threat to human health [2]. Currently, the treatment of LC is dominated by surgery and supplemented by chemotherapy, radiotherapy, targeted therapy, and immunotherapy [3]. Despite multiple strategies and interventions, the prognosis for LC is still unsatisfactory, with a 5-year survival rate of only 50% to 60% in patients and a third of the cases relapsing [4].

For patients with LC, lymph node metastasis (LNM) is one of the most important factors in their treatment and prognosis [5]. In clinical diagnosis and treatment, the palpation of neck nodes using ultrasonography (US), computed tomography (CT), magnetic resonance imaging (MRI), or positron emission tomography-computed tomography (PET-CT) is frequently used to evaluate lymph node status [6, 7]. However, several deficiencies remain with respect to sensitivity and specificity in the abovementioned methods [8]. Previous studies have reported that a large proportion of LC patients diagnosed with LNM preoperatively who underwent intraoperative cervical lymph node dissection (LND) have a negative pathological diagnosis after surgery [9]. Conversely, some patients with clinically negative cervical lymph nodes have positive pathology results after surgery, leading to delayed treatment or even secondary surgery [10]. Therefore, the development of new diagnostic tools for accurately determining preoperative cervical lymph node status is highly essential for selecting appropriate therapy and determining prognosis.

The machine learning (ML) algorithm has been widely used in developing disease prediction models in recent years [11]. Compared with conventional statistical methods, ML can more accurately predict outcomes from multiple unrelated datasets by using high-level computing to construct algorithms for automated data-driven predictions or decisions [12, 13]. Nevertheless, there is no relevant research on the ML to predict the LNM of LC. In this study, we aimed to identify risk factors associated with LNM in LC patients and developed multiple ML-based models for the preoperative prediction of LNM by using clinical and histopathological parameters in Surveillance, Epidemiology, and End Results (SEER) public data. In addition, we chose the best ML model for predicting the risk of LNM in LC patients by comparing the assessment indicators of predictive performance, which aim to identify an accurate prediction method and guide the selection of clinical diagnoses and treatment plans. Meanwhile, the correlation between LNM and clinicopathological characteristics in LC patients was interpreted through the Shapley additive explanation (SHAP) value to help clinicians understand the output of the model.

## 2. Materials and Methods

### 2.1. Study Population

**Figure 1:**
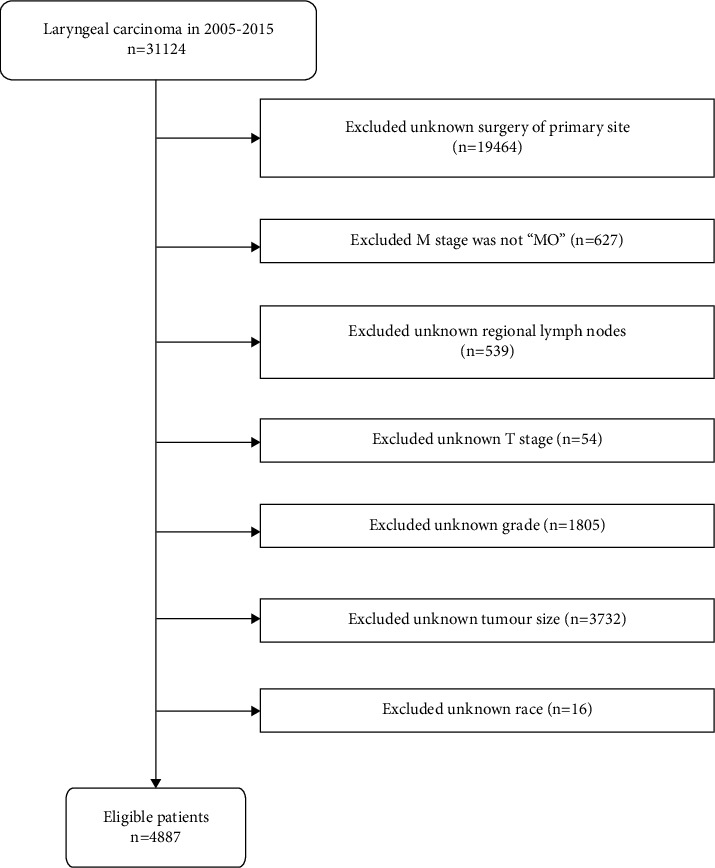
Flowchart of the detailed screening of the study population.

The SEER database, managed by the National Cancer Institute, is a publicly available large population-based cancer registry database that covers almost 30% of the population of the United States [14]. All patient data in this study were downloaded from the SEER database after receiving approval and permission from SEER. Patient data with a confirmed diagnosis of LC were screened from “Incidence-SEER 18 Regs Research Data, Nov 2020 Sub (2000–2018).” The study was limited to the period between 2005 and 2015. The inclusion criteria are as follows: (1) the primary tumour site was in the larynx; (2) the pathological diagnosis was classified as “positive”; (3) the histology of the tumour was categorized as “malignant.” The exclusion criteria are as follows: (1) unknown information about race, T-stage, grade, tumour size, regional lymph nodes, and surgery of the primary site; (2) the M stage was not “M0.” Overall, 4887 patients fulfilled the selection criteria and were chosen for further analysis. The International Classification of Diseases for Oncology, version 3 (ICD-O-3), was used to determine tumour location, grade, and histology. Tumour staging was determined based on the 6th edition of the American Joint Committee on Cancer (AJCC) staging. The patient screening procedure is displayed in Figure 1.

### 2.2. Data Classification

In this study, nine demographics and clinicopathological variables from the SEER database that may affect LNM in LC patients were selected, including age at diagnosis, race, sex, primary site, histology, number of tumours, T-stage, grade, and tumour size.

Patients were divided into groups according to their age at diagnosis: <50 years, 50–65 years, and ≥65 years. Patients were divided into groups according to race: white, black, and others. Patients were classified into groups based on sex: male and female. According to the primary site, the patients were divided as follows: supraglottis, glottis, subglottis, larynx, and others. According to histology, the patients were classified into squamous cell carcinoma and nonsquamous cell carcinoma groups. Based on the number of tumours, the patients were classified into 1 and >1 groups. According to tumour size, the patients were classified into ≤3 cm and >3 cm groups. The grades were categorized as follows: grade I, grade II, grade III, and grade IV. The T-stage was categorized as follows: T1, T2, T3, and T4.

### 2.3. Statistical Analyses

In this study, all statistical analyses were carried out using SPSS (version 22.0, IBM). Patients involved in this study were separated into two groups: nonlymph node metastasis (NLNM) and LNM. The chi-square test was performed to compare the differences between the two groups. A *p* value less than 0.05 was considered to indicate that the identical attributes were significantly different between the two groups. A correlation analysis of all variables was evaluated by the Pearson correlation, and the results are displayed as a heatmap. In addition, univariate and multivariate logistic regression analyses were used to identify independent risk factors for LNM. In the univariate analyses, variables with *p* values less than 0.05 were regarded as statistically significant and chosen for multivariate analyses. Then, variables with *p* values below 0.05 in the multivariable logistic regression analysis were taken as candidate variables for the establishment of ML models.

### 2.4. Model Establishment

For this study, Python software was used to establish the ML models. All patients were divided into a training set (*n* = 3909) and a test set (*n* = 978) at a ratio of 8 : 2 by random sampling, until no significant differences were observed in baseline clinical characteristics (Supplementary Table S1). The training set was used to establish the model, and then the model was validated on the test set. A total of six different ML algorithms were used to model the data in the training set, including logistic regression (LR), k-nearest neighbor (KNN), support vector machine (SVM), extreme gradient boosting (XGBoost), random forest (RF), and light gradient boosting machine (LightGBM). Furthermore, 10-fold cross validation was performed to gauge the stability of the model and determine whether the model was overfitted. Accuracy, precision, recall rate, F1-score, area under the ROC curve (AUC), specificity, and calibration plots were used to evaluate ML models. The Brier score, which ranges from 0 to 1, was used to quantify the calibration plots [15]. The AUCs were compared by the DeLong test, and a *p* value less than 0.05 was considered to indicate that there was a significant difference between the two models.

The Shapley additive explanation (SHAP) framework, a game theory approach, has been used to interpret the output of any ML model [16]. This approach interprets each feature variable by assigning a specific prediction weight and calculating its importance value. In this study, we used the SHAP value to improve the interpretation of the best model [17].

## 3. Results

### 3.1. Demographic and Pathological Characteristics

**Figure 2:**
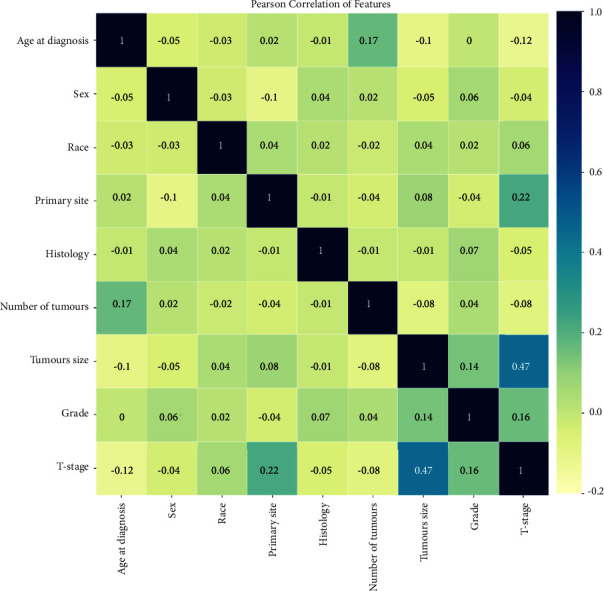
The results of the Pearson correlation analysis between all the variables. The heatmap shows the correlation between the variables.

A total of 4887 cases were available in this study, including 3409 cases without LNM (69.76%) and 1478 cases with LNM (30.24%) (Table 1). In the comparison of the two groups, all variables except histology were considered to be significantly different (*p* < 0.05), including age at diagnosis, race, sex, primary site, number of tumours, T-stage, grade, and tumour size. Details for the groups are summarized in Table 1. In addition, all variables were entered into a Pearson correlation analysis, of which the result shows that variables were weakly correlated with each other and had good independence (Figure 2).

### 3.2. Analysis of Risk Factors for Lymph Node Metastases

Based on the univariate logistic regression analysis, age at diagnosis, race, sex, primary site, number of tumours, T-stage, grade, and tumour size were significantly correlated with LNM in LC patients (*p* < 0.05) (Table 2). Further multivariate logistic regression analysis showed that age at diagnosis, race, primary site, number of tumours, T-stage, grade, and tumour size were identified as independent risk factors for the LNM of LC (Table 3).

### 3.3. Model Performance

**Figure 3:**
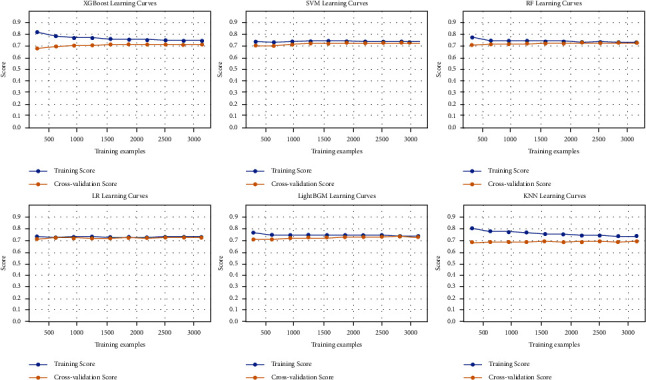
The learning curve for 10-foldcross-validation in the training set. Abbreviations: XGBoost, extreme gradient boosting; SVM, support vector machine; RF, random forest; LR, logistic regression; LightGBM, light gradient boosting machine; KNN, k-nearest neighbor.

**Figure 4:**
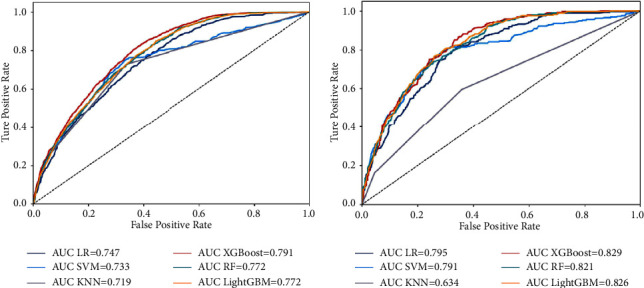
Receiver operating characteristic curves of predictive models based on machine learning algorithms. (a) Training set; (b) test set. Abbreviations: AUC, the area under the receiver operating characteristic curves; XGBoost, extreme gradient boosting; SVM, support vector machine; KNN, k-nearest neighbor; LR, logistic regression; RF, random forest; LightGBM, light gradient boosting machine.

**Figure 5:**
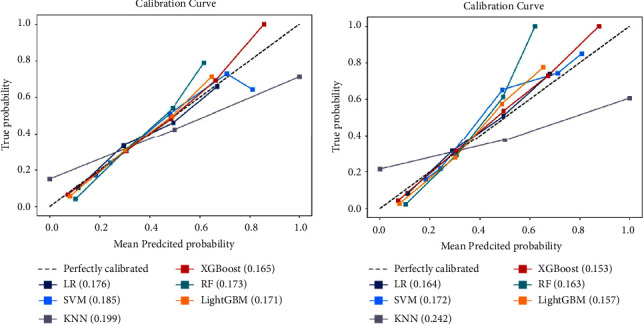
Calibration curves for predicting LNM with various models. The 45 straight line on each graph represents the perfect match between the observed (*y*-axis) and predicted (*x*-axis) survival probabilities. A closer distance between two curves indicates greater accuracy. (a) Training set; (b) test set. Abbreviations: LNM, lymph node metastasis; XGBoost, extreme gradient boosting; SVM, support vector machine; KNN, k-nearest neighbor; LR, logistic regression; RF, random forest; LightGBM, light gradient boosting machine.

In this study, we constructed 6 prediction models by using the 7 aforementioned independent risk factors. As shown in Figure 3, all six models had good stability, and there was no obvious overfitting or underfitting in each model (the performance in 10-fold cross validation was shown in Supplementary Table S2, and the model parameters were shown in Supplementary Table S3). The AUC, F1-score, accuracy, precision, recall rate, specificity, and Brier score were the main evaluation metrics used to evaluate and compare the model performances. However, the higher the value of AUC, F1-score, accuracy, precision, specificity, and recall rate, the better the model performances, but the Brier score was just the opposite [18]. In both the training and test sets, the XGBoost model showed the highest values regarding AUC, F1-score, accuracy, and recall rate, and the lowest values regarding Brier score (AUC = 0.791 (95% CI: 0.776–0.806) and 0.829 (95% CI: 0.818–0.843), F1-score = 0.663 and 0.706, accuracy = 0.739 and 0.770, recall rate = 0.638 and 0.677, Brier score = 0.165 and 0.153) (Figures 4(a), 4(b), 5(a), and 5(b) Table 4). The DeLong test showed a significant difference in the AUC value of the XGBoost model compared with others in the training set (*p* < 0.001), but the AUC value of the XGBoost model had no significant difference compared with both RF and LightGBM models in the test set (XGBoost against RF, *p*=0.151; XGBoost against LightGBM, *p*=0.521). The result of the DeLong test was given in Supplementary Tables S4 and S5. In addition, the precision of SVM (0.696), KNN (0.727), RF (0.713), and LightGBM (0.691) were higher than XGBoost's (0.690) in the training set, but the precision of XGBoost (0.738) was only slightly lower than that of SVM (0.755) and RF (0.745) in the test set (Table 4). In the training and test sets, the specificities of SVM (0.944 and 0.960, respectively), KNN (0.961 and 0.955, respectively), RF (0.956 and 0.969, respectively), and LightGBM (0.916 and 0.925, respectively), were higher than XGBoost (0.894 and 0.912, respectively) (Table 4). Thus, overall, the abovementioned results show that the overall performance of XGBoost is better than other models, so we chose the XGBoost model as the best prediction model.

### 3.4. Feature Importance Analysis

**Figure 6:**
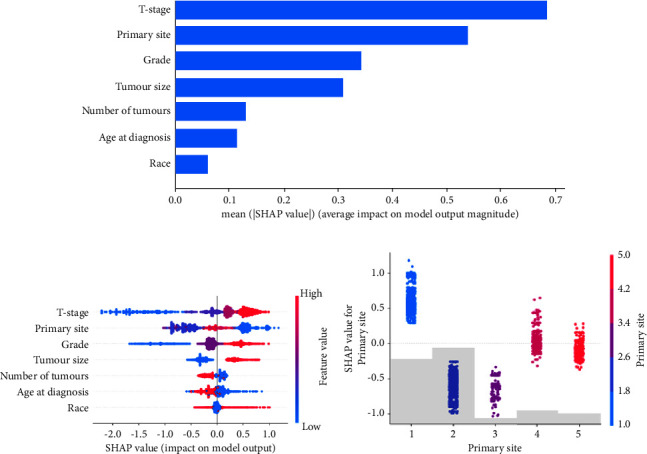
The model's interpretation. (a) Features of importance derived from the XGBoost model. The plot shows the relative importance of the features in the XGBoost model. (b) SHAP summary plot of the 7 risk features of the XGBoost model. The higher the SHAP value of a feature, the higher the probability of LNM development. A dot is created for each feature attribution value for the model of each patient, and thus one patient is allocated one dot on the line for each feature. Dots are colored according to the values of features for the respective patient and accumulate vertically to depict density. Red represents higher feature values, and blue represents lower feature values. (c) SHAP dependence plot of the primary site. The SHAP dependence plot shows how a single feature affects the output of the XGBoost prediction mode. SHAP values for specific features exceed zero, representing an increased risk of LNM. Abbreviations: SHAP, Shapley additive explanation; XGBoost, extreme gradient boosting; LNM, lymph node metastasis.

We further analysed information provided by the XGBoost model about the importance of features. The mean value of the absolute SHAP values of 7 feature variables represents the degree of influence on the final predicted probability (Figure 6(a)); the higher the SHAP value, the stronger the effect of the feature variable on the model output [19]. The SHAP summary plot shows the positive or negative impact of feature variables on the predicted probability through different colors (Figure 6(b)). We found that T-stage, primary site, and grade were the three most important feature variables in the XGBoost model for predicting LNM, and the higher the stage of T-stage and grade, the higher the risk of LNM in patients.

Unlike T-stage and grade, the colour distribution was irregular for the “primary site,” which indicated that the values of the feature were not linearly correlated with the SHAP values (Figure 6(b)). To explore the reason, we further plotted the SHAP dependence plot of this (Figure 6(c)). In the XGBoost model for this study, primary sites 1, 2, 3, 4, and 5 represent “supraglottis,” “glottis,” “subglottis,” “larynx,” and “other,” respectively. Since “larynx” and “other” cannot represent the exact location of the primary tumour anatomically, we concluded that “supraglottis” had a higher risk of LNM than “subglottis” and “glottis.”

## 4. Discussion

LNM is one of the vitally important hallmarks of LC distant metastasis. There is an extensively rich lymphatic vascular network in the neck, which makes it easier for LC patients to present with LNM [20]. Currently, the presence of LNM is mainly determined by cervical palpation and preoperative imaging, which largely depend on the clinical experience of the doctor and the ability of the human eye to identify imaging [21, 22]. As reported, the presence of occult metastasis was confirmed by postsurgery histological examination in 4%–40% of cN0 LC patients [23–25]. Although prophylactic cervical LND can decrease the risk of LNM [26], it puts LC patients at a higher risk for operations, such as major bleeds, lymphatic fistulas, or impairments in the vagus, brachial plexus, and recurrent laryngeal nerve [27]. In the traditional methods for detecting LNM, the sensitivity and specificity of cervical palpation are low, and both false-positive and false-negative rates reach approximately 15%–25% [28]. Although the accuracy of imaging is higher than that of cervical palpation, the nodal size, shape, and presence of central necrosis taken as the criteria to assess LNM status are not reliable [29]. Some enlarged lymph nodes could be mediated by inflammatory hyperplasia, and LNM <10 mm in diameter usually do not exhibit irregular enhancement or central necrosis [30]. Therefore, identifying risk factors associated with LNM and developing a good predictive performance model of LNM for LC is extremely important for clinicians to select appropriate treatment and improve the prognosis of patients.

In this study, we found that more than 90% of LC patients were over 50 years old or had squamous cell carcinoma (Table 1), which was consistent with some previous studies [31]. Moreover, LNM was more common among males than females (Table 1), which matches the overall sex-based incidence of LC and may be associated with higher smoking and drinking rates in males [32]. Mutlu et al. found that the incidence of cervical metastasis occurring in supraglottic tumours was significantly higher than that in transglottic tumours (55.2% and 35.1%, respectively) [33], which was similar to our results that the occurrence proportion of LNM was highest in the supraglottic region (Table 1). The abovementioned results indicate that the cases included in our study are in accordance with the epidemiological characteristics of LC, with good representation.

In addition, we also found that age at diagnosis, race, primary site, number of tumours, tumour size, grade, and T-stage were closely associated with the occurrence of LNM of LC by univariate and multivariate logistic regression analyses (Tables 2 and 3). Among them, T-stage, grade, and tumour size have been previously reported as independent risk factors for LNM in LC patients, which is consistent with our study findings, indicating that these variables play an important role in promoting LNM in LC patients [34]. Our multivariate logistic regression analysis further demonstrated that the primary site was a risk factor for LNM (Table 3). This suggested that the primary tumour site was strongly associated with LNM, which may be related to the supraglottic special anatomy that contains an abundant and extensive submucosal lymphatic plexus [35]. Furthermore, similar to previous studies evaluating the risk factors for LNM in head and neck tumours [8], LC patients with a younger age at diagnosis presented a higher risk of LNM in this study. Interestingly, a single tumour presents a significantly higher risk of LNM than multiple tumours in our study. Multifocal tumours have been considered a feature with more aggressiveness and were more likely to develop LNM than single tumours, which is contrary to the results of our findings [36]. However, the reason for the difference remains unclear and will require further study.

In recent years, various risk factors for LNM of LC have been reported, and prediction models have been established [8, 34]. However, due to the complexity and large size of the various factors in the data and the differences among the calculation methods of the models, the prediction performance was also significantly different. Heng et al. [37] developed a nomogram to predict the occult lymph node metastases of glottic squamous cells with an AUC value of 0.716. Chen et al. [34] used a nomogram to predict cervical LNM in laryngeal squamous cell carcinoma with an AUC value of 0.809. Furthermore, Song et al. used a nomogram to predict the risk of LNM in newly diagnosed supraglottic laryngeal squamous cell carcinoma, in which the AUC value of the nomogram model was only 0.731 and 0.707 in their training set and test set, respectively [8]. All three studies used the same logic calculation method, but the prediction performance of the models varied widely. To more accurately predict LNM in LC patients, we established prediction models using six different ML algorithms for the first time and selected the prediction model with the best performance by comparing the performance differences among various prediction models. The accuracy, precision, recall rate, F1-score, AUC value, specificity, and calibration plots were performed to evaluate and compare the ML model performances, and we found that XGBoost was better than the other models with respect to the AUC value, Brier score, F1-score, recall rate, and accuracy, whether it was in the training set or in the test set (Table 4, Figures 4(a), 4(b), 5(a), and 5(b)). In addition, the AUC value of XGBoost was also higher than that of the model constructed in previous studies [8, 34, 37]. Therefore, we believe that XGBoost was the best predictive model to predict the LNM in LC patients.

XGBoost, as an ensemble ML algorithm based on a decision tree, has the advantages of fast computation, maximizing predictive performance, minimizing model complexity, and low overfitting [38]. Therefore, XGBoost has been widely used in prediction model construction and risk identification in the medical field [39]. However, this algorithm also has the following shortcomings. First, there are too many hyperparameters in this algorithm, which would greatly influence the training time and performance of the XGBoost model [40], and secondly, this algorithm is difficult to visualize and explain models, which results in the limitation of the ML model in daily applications to some extent [41]. Based on the abovementioned features, the SHAP method was introduced to explain the importance and contribution of variables in the XGBoost model to help more clinicians understand the mechanism behind ML. We found that T-stage, primary site, and grade were the three most important feature variables in the XGBoost model for predicting LNM, and race was the least important variable (Figure 6(a)). In addition, given that the values of the primary site feature were not linearly correlated with the SHAP values in the SHAP summary plot (Figure 6(b)), the additional SHAP dependence plot revealed that the supraglottis was an important feature leading to LNM, whereas the subglottis and glottis were the opposite (Figure 6(c)). This finding was consistent with previous studies showing that the supraglottic type of LC is more likely to have LNM [42].

Although T-stage and primary site are important bases for preoperative judgement of LNM at present, it remains controversial to perform cervical LND only on these bases. The National Comprehensive Cancer Network (NCCN) considered that all patients with supraglottic lesions or T3-T4 stages should have neck LNM [43]. However, a study by Sessions et al. found that patients with N0 disease may be safely observed with no loss of survival advantage [44]. Furthermore, Ömer et al. showed that a watchful waiting strategy can be applied to T1-T2 and selected T3 cases with well-differentiated tumours [23]. These data further suggested that the LNM of LC is influenced by several factors, and that it is not accurate to judge LNM solely by T-stage and primary site. Based on our XGBoost model, the individual risk of LNM can be identified more accurately, and the LND strategies for LC patients can also be determined by doctors directly and accurately, thereby avoiding overtreatment and reducing the risk of complications related to neck dissection. Some limitations exist in this study. First, our study is a retrospective study and suffers from some possible selection bias. Second, since the patients in the study were predominantly from the North American population, there may be deficiencies in the applicability of the population, so including a wider population is necessary for future research. Finally, all patients in this study were from a single database, and a multicentre study is required for external validation of our model.

## 5. Conclusion

In this study, we found that age at diagnosis, race, primary site, number of tumours, tumour size, grade, and T-stage were independent risk factors for LNM of LC. In addition, we developed six ML models to predict LNM in LC patients based on this information. All models performed well, but the XGBoost model had better predictive power. Finally, through the SHAP method, we determined that T-stage, primary site, and grade were the three most important feature variables in the XGBoost model for predicting LNM. In the future, we will validate our findings through a prospective multicenter study using a completely independent external dataset.

## Figures and Tables

**Table 1:** Clinical and pathological characteristics features of patients.

Variables	NLNM	LNM	*p* value
	3409 (69.76%)	1478 (30.24%)	
Age at diagnosis			*p* < 0.001
≥65	1653 (48.49%)	548 (37.08%)	
50–65	1643 (48.20%)	874 (59.13%)	
<50	113 (3.31%)	56 (3.79%)	

Race			*p*=0.002
White	2764 (81.08%)	1132 (76.59%)	
Black	502 (14.73%)	267 (18.06%)	
Other	143 (4.19%)	79 (5.35%)	

Sex			*p*=0.046
Male	2817 (82.63%)	1186 (80.24%)	
Female	592 (17.37%)	292 (19.76%)	

Primary site			*p* < 0.001
Supraglottis	1027 (30.13%)	824 (55.75%)	
Glottis	1829 (53.65%)	346 (23.41%)	
Subglottis	128 (3.75%)	36 (2.44%)	
Larynx	238 (6.98%)	152 (10.28%)	
Other	187 (5.49%)	120 (8.12%)	

Histology			*p*=0.522
Squamous cell carcinoma	3264 (95.75%)	1421 (96.14%)	
Nonsquamous cell carcinoma	145 (4.25%)	57 (3.86%)	

Number of tumours			*p*=0.001
1	2081 (61.04%)	1021 (69.08%)	
>1	1328 (38.96%)	457 (30.92)	

Tumour size			*p*=0.001
≤3 cm	2429 (71.25%)	601 (40.66%)	
>3 cm	980 (28.75%)	877 (59.34%)	

Grade			*p* < 0.001
Grade I	575 (16.87%)	63 (4.26%)	
Grade II	2055 (60.28%)	783 (52.98%)	
Grade III and IV	779 (22.85%)	632 (42.76%)	

T-stage			*p* < 0.001
T1	1185 (34.76%)	100 (6.77%)	
T2	589 (17.28%)	229 (15.49%)	
T3	648 (19%)	393 (26.59%)	
T4	987 (28.95%)	756 (51.15%)	

Notes: chi-square tests were compared between the two groups (*p* < 0.05 represents a statistically significant difference). Abbreviations: LNM, lymph node metastasis; NLNM, nonlymph node metastasis.

**Table 2:** A univariate logistic regression analysis of variables related to LNM.

Variables	OR	95% CI	*p* value
Age at diagnosis			
≥65	Reference		
50–65	1.495	1.070–2.089	0.019
50	1.605	1.414–1.821	<0.001

Race			
White	Reference		
Black	1.299	1.102–1.530	0.002
Other	1.349	1.016–1.791	0.038

Sex			
Male	Reference		
Female	1.172	1.003–1.369	0.046

Primary site			
Supraglottis	Reference		
Glottis	0.236	0.204–0.273	<0.001
Subglottis	0.351	0.239–0.513	<0.001
Larynx	0.796	0.637–0.995	0.045
Other	0.80	0.625–1.024	0.076

Histology			
Squamous cell carcinoma	Reference		
Nonsquamous cell carcinoma	0.903	0.66–1.234	0.522

Number of tumours			
1	Reference		
>1	0.701	0.616–0.799	<0.001

Tumour size			
≤3 cm	Reference		
>3 cm	3.617	3.184–4.109	<0.001

Grade			
Grade I	Reference		
Grade II	3.478	2.647–4.568	<0.001
Grade III and IV	7.405	5.594–9.802	<0.001

T-stage			
T1	Reference		
T2	4.607	3.571–5.945	<0.001
T3	7.187	5.656–9.132	<0.001
T4	9.077	7.248–11.367	<0.001

Notes: *p* < 0.05 represents a statistically significant difference. Abbreviations: LNM, lymph node metastasis.

**Table 3:** Multivariate logistic regression analysis of variables related to LNM.

Variables	OR	95% CI	*p* value
Age at diagnosis			
≥65	Reference		
50–65	1.354	0.931–1.970	0.113
<50	1.248	1.080–1.442	0.003

Race			
White	Reference		
Black	1.064	0.886–1.277	0.510
Other	1.538	1.109–2.133	0.010

Sex			
Male	Reference		
Female	0.939	0.784–1.123	0.489

Primary site			
Supraglottis	Reference		
Glottis	0.289	0.252–0.354	<0.001
Subglottis	0.254	0.169–0.381	<0.001
Larynx	0.522	0.408–0.669	<0.001
Other	0.511	0.390–0.670	<0.001

Number of tumours			
1	Reference		
>1	0.744	0.642–0.863	<0.001

Tumour size			
≤3 cm	Reference		
>3 cm	1.742	1.498–2.026	<0.001

Grade			
Grade I	Reference		
Grade II	2.438	1.818–3.267	<0.001
Grade III and IV	4.609	3.407–6.235	<0.001

T-stage			
T1	Reference		
T2	2.923	2.231–3.829	<0.001
T3	3.925	3.030–5.084	<0.001
T4	5.890	4.571–7.590	<0.001

Notes: *p* < 0.05 represents a statistically significant difference. Abbreviations: LNM, lymph node metastasis.

**Table 4:** Comparison and prediction performances of different models for LNM.

Models	AUC (95% CI)	Accuracy	Precision	F1-score	Recall-rate	Specificity
Training set						
XGBoost	0.791 (0.776–0.806)	0.739	0.690	0.663	0.638	0.894
SVM	0.733 (0.707–0.750)	0.732	0.696	0.640	0.593	0.944
KNN	0.719 (0.702–0.737)	0.738	0.727	0.653	0.592	0.961
LR	0.747 (0.733–0.763)	0.728	0.671	0.646	0.623	0.888
RF	0.772 (0.757–0.787)	0.734	0.713	0.645	0.589	0.956
LightGBM	0.772 (0.757–0.787)	0.736	0.691	0.653	0.619	0.916

Test set						
XGBoost	0.829 (0.818–0.843)	0.770	0.738	0.706	0.677	0.912
SVM	0.791 (0.778–0.805)	0.755	0.755	0.681	0.620	0.960
KNN	0.634 (0.618–0.653)	0.715	0.666	0.607	0.558	0.955
LR	0.795 (0.780–0.808)	0.748	0.706	0.675	0.647	0.905
RF	0.821 (0.808–0.833)	0.740	0.745	0.659	0.591	0.969
LightGBM	0.826 (0.813–0.839)	0.759	0.729	0.687	0.650	0.925

Abbreviations: XGBoost, extreme gradient boosting; SVM, support vector machine; KNN, k-nearest neighbor; LR, logistic regression; RF, random forest; LightGBM, light gradient boosting machine; LNM, lymph node metastasis.

## Data Availability

The data included and analysed in this study are available in the SEER database (https://seer.cancer.gov/). Our implementation is based on Python 3.9. More specifically, the ML model was built by using the Python package “scikit learn 1.1.1” (https://scikit-learn.org/stable/) and “SciPy 1.7.3” (https://www.scipy.org/); SHAP was based on “shap 0.39.0” (https://shap.readthedocs.io/en/latest/).

## Supplementary Materials

Supplementary Materials Supplementary Table S1: lymph node metastasis ratio in both training and test sets. Supplementary Table S2: the performance of different models for LNM in 10-foldcross-validation. Supplementary Table S3: the parameters used to develop models. Supplementary Table S4: DeLong test of the ROC curve in the training set. Supplementary Table S5: DeLong test of the ROC curve in the test set.
