# Supplementary material for: Application of an Interpretable Machine Learning Model to Predict Lymph Node Metastasis in Patients with Laryngeal Carcinoma
Source: J Oncol. 2022 Nov 12;2022:6356399. doi: 10.1155/2022/6356399 (PMC9675609; doi:10.1155/2022/6356399)
Supplement: Supplementary Materials — Supplementary Table S1: lymph node metastasis ratio in both training and test sets. Supplementary Table S2: the performance of different models for LNM in 10-foldcross-validation. Supplementary Table S3: the parameters used to develop models. Supplementary Table S4: DeLong test of the ROC curve in the training set. Supplementary Table S5: DeLong test of the ROC curve in the test set. [file 6356399.f1.zip › Supplementary Table 1.doc]

Table S1 Lymph nodes metastasis ratio in both training and test sets.

|  | NLNM | LNM |
| --- | --- | --- |
| Training set (3909) | 2727(69.76%) | 1182(30.24%) |
| Test set (978) | 682(69.73%) | 296(30.27%) |

Abbreviations: NLNM, non-lymph nodes metastasis; LNM, lymph nodes metastasis.
