# Supplementary material for: Application of an Interpretable Machine Learning Model to Predict Lymph Node Metastasis in Patients with Laryngeal Carcinoma
Source: J Oncol. 2022 Nov 12;2022:6356399. doi: 10.1155/2022/6356399 (PMC9675609; doi:10.1155/2022/6356399)
Supplement: Supplementary Materials — Supplementary Table S1: lymph node metastasis ratio in both training and test sets. Supplementary Table S2: the performance of different models for LNM in 10-foldcross-validation. Supplementary Table S3: the parameters used to develop models. Supplementary Table S4: DeLong test of the ROC curve in the training set. Supplementary Table S5: DeLong test of the ROC curve in the test set. [file 6356399.f1.zip › Supplementary Table 2.doc]

Table S2 The performance of different models for LNM in 10-fold cross-validation.

|  | Mean | Mean | Mean | Mean | Mean | Mean |
| --- | --- | --- | --- | --- | --- | --- |
|  | AUC(SD) | Accuracy(SD) | F1-score(SD) | Recall-rate(SD) | Precision(SD) | Brier-score(SD) |
| LR | 0.744(0.017) | 0.727(0.019) | 0.439(0.046) | 0.356(0.043) | 0.531(0.042) | 0.180(0.008) |
| SVM | 0.716(0.025) | 0.724(0.020) | 0.321(0.055) | 0.218(0.044) | 0.526(0.035) | 0.189(0.010) |
| KNN | 0.626(0.024) | 0.702(0.019) | 0.264(0.053) | 0.178(0.039) | 0.389(0.035) | 0.255(0.014) |
| XGBoost | 0.758(0.017) | 0.724(0.019) | 0.442(0.031) | 0.363(0.040) | 0.537(0.033) | 0.176(0.009) |
| RF | 0.761(0.018) | 0.728(0.022) | 0.318(0.035) | 0.210(0.026) | 0.551(0.034) | 0.176(0.009) |
| LightGBM | 0.764(0.021) | 0.732(0.017) | 0.412(0.038) | 0.313(0.042) | 0.552(0.032) | 0.173(0.009) |

Abbreviations: LR, logistic regression; SVM, support vector machine; KNN, k-nearest neighbor; XGBoost, extreme gradient boosting; RF, random forest; LightGBM, light gradient boosting machine; SD, standard deviation
