# Supplementary material for: Application of an Interpretable Machine Learning Model to Predict Lymph Node Metastasis in Patients with Laryngeal Carcinoma
Source: J Oncol. 2022 Nov 12;2022:6356399. doi: 10.1155/2022/6356399 (PMC9675609; doi:10.1155/2022/6356399)
Supplement: Supplementary Materials — Supplementary Table S1: lymph node metastasis ratio in both training and test sets. Supplementary Table S2: the performance of different models for LNM in 10-foldcross-validation. Supplementary Table S3: the parameters used to develop models. Supplementary Table S4: DeLong test of the ROC curve in the training set. Supplementary Table S5: DeLong test of the ROC curve in the test set. [file 6356399.f1.zip › Supplementary Table 3.doc]

Table S3 The parameters used to develop models.

| model | parameters |
| --- | --- |
| LR | penalty='l2',dual=False, tol=0.0001, C=1.0, , max_iter=100 |
| SVM | C=0.5, kernel='rbf', degree=6, gamma='auto', coef0=0.0 |
| KNN | k=2, weights='uniform', leaf_size=5, p=2 |
| XGBoost | random_state=50, max_depth=3, learning_rate=0.2, n_estimators=100 |
| RF | random_state=50, max_depth=4, n_estimators=200 |
| LightGBM | random_state=50, max_depth=2, n_estimators=50, learning_rate=0.1 |

Abbreviations: LR, logistic regression; SVM, support vector machine; KNN, k-nearest neighbor; XGBoost, extreme gradient boosting; RF, random forest; LightGBM, light gradient boosting machine; SD, standard deviation.
