# Supplementary material for: Application of an Interpretable Machine Learning Model to Predict Lymph Node Metastasis in Patients with Laryngeal Carcinoma
Source: J Oncol. 2022 Nov 12;2022:6356399. doi: 10.1155/2022/6356399 (PMC9675609; doi:10.1155/2022/6356399)
Supplement: Supplementary Materials — Supplementary Table S1: lymph node metastasis ratio in both training and test sets. Supplementary Table S2: the performance of different models for LNM in 10-foldcross-validation. Supplementary Table S3: the parameters used to develop models. Supplementary Table S4: DeLong test of the ROC curve in the training set. Supplementary Table S5: DeLong test of the ROC curve in the test set. [file 6356399.f1.zip › Supplementary Table 5.doc]

Table S5 Delong test of ROC curve in test set.

| Model |  |  |  |  |  |
| --- | --- | --- | --- | --- | --- |
| KNN | P<0.0001 |  |  |  |  |
| SVM | P=0.6913 | P<0.0001 |  |  |  |
| RF | P=0.001 | P<0.0001 | P=0.001 |  |  |
| XGBoost | P=0.0002 | P<0.0001 | P<0.0001 | P=0.1508 |  |
| LightGBM | P=0.0001 | P<0.0001 | P<0.0001 | P=0.1196 | P=0.5211 |
|  | LR | KNN | SVM | RF | XGBoost |

Notes: p <0.05 represents a statistically significant difference.

Abbreviations: LR, logistic regression; SVM, support vector machine; KNN, k-nearest neighbor; XGBoost, extreme gradient boosting; RF, random forest; LightGBM, light gradient boosting machine; SD, standard deviation.
